# Correlation of Cerebral Microbleed Distribution to Amyloid Burden in Patients with Primary Intracerebral Hemorrhage

**DOI:** 10.1038/srep44715

**Published:** 2017-03-17

**Authors:** Hsin-Hsi Tsai, Li-Kai Tsai, Ya-Fang Chen, Sung-Chun Tang, Bo-Ching Lee, Ruoh-Fang Yen, Jiann-Shing Jeng

**Affiliations:** 1 Departments of Neurology, National Taiwan University Hospital, Taipei, Taiwan; 2 Department of Medical Imaging, National Taiwan University Hospital, Taipei, Taiwan; 3 Department of Nuclear Medicine, National Taiwan University Hospital, Taipei, Taiwan

## Abstract

The underlying pathology of cerebral microbleeds (CMBs) with mixed lobar and deep distribution remains contentious. The aim of this study was to correlate CMBs distribution to β-amyloid burden in patients with primary intracerebral hemorrhage (ICH). Fourty-seven ICH patients underwent magnetic resonance susceptibility-weighted imaging and ^11^C-Pittsburgh Compound B positron emission tomography. The amyloid burden was expressed as standardized uptake value ratio with reference to cerebellum, and presented as median (interquartile range). Patients were categorized into the lobar, mixed (both lobar and deep regions), and deep types of CMB. Comparing the lobar (17%), mixed (59.6%) and deep (23.4%) CMB types, the global amyloid burden was significantly higher in the mixed type than the deep type (1.10 [1.03–1.25] *vs* 1.00 [0.97–1.09], *p* = 0.011), but lower than in the lobar type (1.48 [1.18–1.50], *p* = 0.048). On multivariable analysis, the ratio of lobar to deep CMB number was positively correlated with global (*p* = 0.028) and occipital (*p* = 0.031) amyloid burden. In primary ICH, patients with lobar and mixed CMB types are associated with increased amyloid burden than patients with deep type. The ratio of lobar to deep CMB number is an independent indicator of cerebral β-amyloid deposition.

Intracerebral hemorrhage (ICH) accounts for about one-fifth of all strokes and its incidence has not decreased since 1980^1^. About 80% of patients are classified as having primary ICH, with hypertensive angiopathy (HA) and cerebral amyloid angiopathy (CAA) accounting for the majority of cases^2^,^3^. Differentiation between HA and CAA is traditionally based on hemorrhage location^3^,^4^. CAA-related hemorrhage is typically located in the lobar region and hypertensive ICH is usually located in the deep region^5^,^6^. However, because hypertension also causes lobar hemorrhage^7^, and the possibility of concomitant HA and CAA^4^,^8^, the pathogenesis of ICH is sometimes difficult to ascertain.

Cerebral microbleeds (CMBs) can be detected by using magnetic resonance (MR) gradient-echo T2* or susceptibility-weighted imaging (SWI)^9^. The different patterns of CMB distribution might reflect different vascular pathologies^9^. The presence of an exclusively lobar distribution of CMBs is highly correlated with advanced CAA^10^,^11^. However, CMBs with mixed lobar and deep distributions are frequently encountered in clinical practice, especially in Asian populations^12^. Whether the hemorrhage in a patient with mixed CMB distribution is attributed to HA, CAA, or other small vessel disease cannot be fully confirmed because of the lack of a standardized diagnostic tool in addition to pathological analysis.

The application of amyloid positron emission tomography (PET) using ^11^C-Pittsburgh Compound B (PiB) provides a noninvasive method for identifying β-amyloid burden in CAA patients^13^,^14^,^15^. PiB retention shows regional association with microhemorrhages and new CAA-related hemorrhages^16^. The introduction of PiB PET can be a tool for CAA diagnosis^17^. In the current study, we used SWI to analyze the CMB distribution and PiB PET to measure the cerebral β-amyloid deposition in ICH patients. We aimed to correlate CMB distribution patterns to cerebral β-amyloid burden, with emphasis on mixed lobar and deep distribution of CMBs.

## Methods

### Subjects

Approximately 250 patients were diagnosed with ICH in National Taiwan University Hospital each year (HA, 54.9%; CAA, 12.2%)^4^. Patients aged older than 20 years with symptomatic primary ICH were recruited from September 2014 to October 2016. We excluded patients with potential causes of hemorrhage including trauma, structural lesion, brain tumor, or coagulopathy due to systemic disease or medication; those who had infarct with hemorrhagic transformation, and those with simultaneous lobar and deep ICHs, or repeated ICHs in both lobar and deep locations. Patients who could not receive the PET and MRI studies, such as poor cooperation, hemodynamic instability, and implantation of cardiac pacemaker were also excluded. The clinical diagnosis of ICH was based on the SMASH-U criteria^3^,^4^. Control subjects without dementia or neurological disease were included to evaluate normal cerebral amyloid deposition.

### Standard Protocol Approvals, Registrations, and Patients Consents

This study was performed with the approval of and in accordance with the guidelines of the institutional review board (201404065MIND) in National Taiwan University Hospital and with the informed consent of all subjects or their family members.

### Imaging acquisition

All subjects underwent a brain MRI with SWI for detection of CMBs. In addition, a ^11^C-PiB PET scan was performed to evaluate brain amyloid deposition. Patients underwent assessment using the Mini-Mental Status Examination (MMSE) and Clinical Dementia Rating Scale prior to PET scanning. The Clinical Dementia Rating score was evaluated mainly on the basis of reports from family members about the patient’s premorbid cognitive decline.

MRI was performed using a 3T MRI scanner (Siemens Verio, TIM, or mMR). SWI acquisition was performed with a T2*-weighted gradient echo sequence with flip angle 15°, TR/TE = 28/20 ms, matrix number = 221 × 320, FOV = 23 cm, slice thickness = 1.6 mm. SWI and minimum intensity projection images were acquired by in-line post-processing of magnitude and phase images, and these post-processed images were used for the evaluation of the imaging findings. Other conventional imaging protocols included T1-weighted three-dimensional magnetization-prepared rapid gradient-echo anatomic imaging, and T1-weighted, T2-weighted, and fluid-attenuated inversion recovery (FLAIR) imaging. Approximately 40 minutes after injection of 10 mCi ^11^C-PiB, static PET images (Discovery ST, GE Healthcare, Milwaukee, WI, USA) were acquired for 30 minutes, and the PET data were reconstructed with ordered set expectation maximization and corrected for attenuation.

### Image analysis

All MRI scans were evaluated by reader H.-H.T. and B.-C.L. independently. If there was disagreement between the 2 readers, a consensus decision was made after discussion. CMBs (defined as lesions with homogeneous round signal loss [less than 10 mm in diameter] on SWI but not as symmetric hypointensities and flow voids from blood vessels) were counted in the lobar region (i.e., the frontal, temporal, parietal, occipital, and insular cortices) and deep region (i.e, the brainstem, cerebellum, basal ganglia, thalamus, internal capsule, external capsule, corpus callosum, and deep periventricular white matter) using the Microbleed Anatomical Rating Scale^18^. The CMB ratio was defined as the number of lobar CMBs divided by the number of deep CMBs. The distribution pattern of CMBs was classified as lobar (no CMBs in the deep region), mixed (CMBs in both the lobar and deep regions) or deep. In addition to CMBs, white matter hyperintense lesions were scored on FLAIR images using the Fazekas scale^19^. Cortical superficial siderosis (cSS) was defined as linear residues of chronic blood products in the superficial layers of the cerebral cortex showing a characteristic gyriform pattern of low signal on SWI^20^. Enlarged perivascular space (EPVS) were defined as sharply delineated structures on T2-weighted imaging, measuring <3 mm following the course of perforating or medullary vessels^21^. The number of EPVS (one side of the brain with more severe involvement) was measured in basal ganglia and centrum semiovale respectively.

Each PiB PET image was realigned, resliced, and manually coregistered to a standardized CT or T1-weighted MR template using PMOD software. Regions of interest on these spatially normalized images were defined using the Automated Anatomical Labeling atlas, with care not to include areas of hemorrhage. The regions of interest included the frontal, temporal, parietal, and occipital lobes. The cerebellar cortex was selected as the reference region because of its low or absent PiB binding. The PET data were semiquantitatively analyzed and expressed as standardized uptake value ratios (SUVRs) of regions of interest. The global neocortical standardized uptake value ratio was also calculated.

### Defining Normal Amyloid Deposition

Fourteen non-demented normal controls (mean age, 55.8 ± 8.6 years; male, 42.9%; MMSE, 29.5 ± 1.2) were included for evaluation of cerebral amyloid deposition. The mean PiB SUVR in these patients was 1.01 ± 0.07. A positive PiB scan was defined as SUVR > 1.15, which was the mean SUVR plus 2 standard deviation (SD) in the control subjects.

### Statistical analysis

Categorical variables are presented as percentages, and the continuous or discrete variables are presented as mean ± SD. The amyloid burden, expressed as SUVR, is presented as median (interquartile range). The characteristics between different subject groups were compared using the Fisher’s exact test for categorical variables and the Kruskal-Wallis test or the Mann-Whitney test for continuous variables. Lobar and deep CMB count, CMB ratio, CMB distribution, age, hypertension, ICH days, and ICH location were examined using multivariable linear regression analysis with global or regional amyloid burden as the dependent variable and using logistic regression analysis with positive PiB scan as the dependent variable. The linear weighted kappa statistic was calculated to evaluate the intra- agreement and inter-observer observer reproducibility of the CMB count. The diagnostic sensitivity, specificity, positive predictive value (PPV), negative predictive value (NPV) and the area under the receiver operating (ROC) curve (Az) of CMBR were also calculated. For determination of the sensitivity and specificity values, CMBR > 2 was considered to be positive, and CMBR ≤ 2 was considered to be negative. All statistical analyses were performed using SPSS version 22 (SPSS Inc., Chicago, IL). All tests of significance were 2-tailed with a threshold for significance of *p* < 0.05.

## Results

### Demographics and Clinical Characteristics

A total of 57 ICH patients (mean age, 65.7 ± 13.4 years; male, 57%) were recruited and 10 patients (17.5%) with negative CMB (global amyloid burden 1.06 [1.00–1.13]) were excluded from further analysis. Twenty-five (53.2%) of the remaining 47 patients had lobar ICH and 22 (46.8%) had deep ICH. The median time of PET acquisition after ICH event was 277 (range 8–3649) days. The interval between the acquisition of MRI and PET scans were within 3 months in all the patients (median 0, range 0–79 days). According to the SMASH-U criteria, 20 patients were classified as CAA (42.6%), 19 patients as HA (40.4%), and 8 patients as undermined etiology (17%). All patients had a premorbid Clinical Dementia Rating score below 0.5. The MMSE results were unavailable in 5 patients because of the appearance of aphasia after ICH. The mean MMSE score for the other 42 patients was 23.8 ± 6.8. The global PiB SUVR was 1.09 (1.01–1.26). There was no intralobar difference in regional PiB SUVR (*p* = 0.118).

The CMB distribution pattern in the 47 patients with CMBs was lobar in 8 patients (17.0%; lobar CMB count, 11.4 ± 14.2, range 1–40), deep in 11 patients (23.4%; deep CMB count, 2.0 ± 1.3, range 1–5), and mixed in 28 patients (59.6%; lobar CMB count, 13.8 ± 15.5, range 1–58; deep CMB count, 11.0 ± 12.0, range 1–49). The intraobserver and interobserver reliability (κ) was 0.84 and 0.70 in the determination of lobar CMB count and 0.76 and 0.72 in the determination of deep CMB count.

The demographics and clinical characteristics are summarized in Table 1. Patients with strictly lobar CMBs were significantly older than those with mixed (79.9 ± 12.5 years *vs* 65.9 ± 12.3 years, *p* = 0.013) or deep (58.5 ± 14 years, *p* = 0.006) CMBs. There was no significant difference in the rate of hypertension, diabetes, or dyslipidemia among these 3 groups. Lobar CMBs were more likely associated with lobar ICH than with mixed or deep CMBs (87.5%, 53.6%, and 27.3%, *p* = 0.035). Based on the SMASH-U criteria, lobar CMBs were most frequently associated CAA than mixed or deep CMBs (75%, 42.9% and 18.2%, *p* = 0.019). The lobar CMB number was similar in patients with lobar and mixed CMBs (*p* = 0.434), but the deep CMB number was higher in patients with mixed CMBs than those with deep CMBs (*p* = 0.001). There was no difference in the comparison of other MRI markers, including white matter score, cSS or EPVS (Supplementary Table 1).

### The Relationship Between CMB Distribution and Amyloid Burden

**Figure 1:**
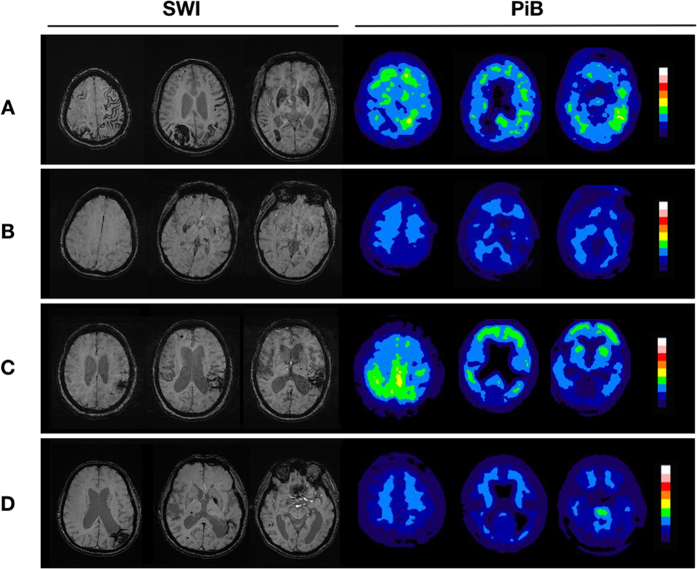
Image findings in patients with primary intracerebral hemorrhage. (**A**) A 94-year-old man with right parietal lobe hemorrhage. SWI shows cortical superficial siderosis and multiple cerebral microbleeds (CMBs) exclusively in the lobar regions (lobar type). Increased amyloid burden is shown on the Pittsburgh compound B (PiB) PET. (**B**) A 57-year-old woman with left thalamic hemorrhage. SWI shows a few CMBs at basal ganglia and thalamus (deep type). PET shows normal amyloid burden. (**C**) A 82-year-old woman with left parietal hemorrhage. SWI shows some CMBs in the lobar region and a few CMBs in the thalamus and basal ganglia (mixed type; CMB ratio = 6/3). PET shows increased PiB uptakes, especially on the occipital lobes. (**D**) A 73-year-old man with left parietal ICH. The SWI shows CMB in both lobar and deep regions (mixed type; CMB ratio = 9/10). PET shows normal amyloid burden.

**Figure 2:**
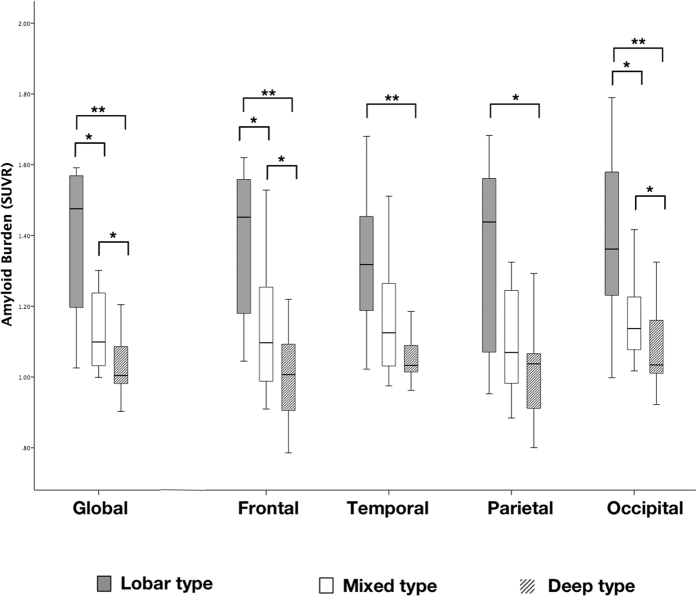
The global and regional amyloid burden in different microbleed patterns. Box plot showing the median values and interquartile ranges of the standardized uptake value ratio (SUVR) representing the global and regional (frontal, temporal, parietal and occipital lobes) amyloid burden in patients with different patterns of cerebral microbleeds. *p < 0.05; **p < 0.01.

Representative SWI and PiB PET images from patients with different patterns of CMB distribution are shown in Fig. 1. The global and regional (frontal, temporal, parietal, and occipital) PiB uptakes were significantly higher in patients with lobar CMBs than those with deep CMBs (Global 1.48 [1.18–1.57] *vs* 1.00 [0.97–1.09], *p* = 0.002) (Fig. 2). In addition, patients with mixed CMBs had higher global PiB uptake than patients with deep CMBs (1.10 [1.03–1.25] *vs* 1.00 [0.97–1.09], *p* = 0.011), lower global PiB uptake than patients with lobar CMBs (1.48 [1.18–1.5], *p* = 0.048), higher regional PiB retention than patients with deep CMBs, and lower regional PiB retention than those with lobar CMBs in the frontal (1.10 [1.00–1.28] vs 1.01 [0.90–1.12] and 1.45 [1.18–1.57], *p* < 0.05) and occipital lobes (1.14 [1.09–1.23] vs 1.03 [1.01–1.16] and 1.36 [1.23–1.58], *p* < 0.05), but not in the temporal and parietal lobes (Fig. 2).

**Figure 3:**
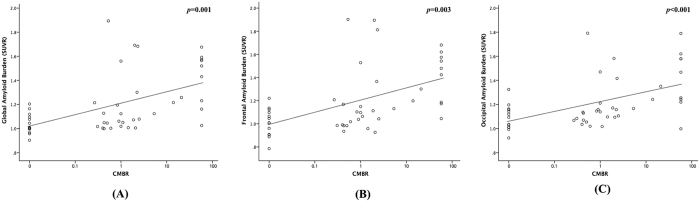
Correlation of cerebral microbleeds ratio and amyloid burden. The correlation of cerebral microbleed (CMB) ratio to the global PiB standardized uptake value ratio (SUVR) (**A**), frontal PiB SUVR (**B**) and occipital PiB SUVR (**C**).

The CMB ratio was calculated as the ratio of the lobar CMB count to the deep CMB count. The patients with lobar CMBs and no deep CMBs had a CMB ratio of 58, which was the highest lobar CMB count observed in our patients. In univariable analysis, the CMB ratio showed a positive correlation to the global, frontal, and occipital PiB uptakes (Fig. 3). On the other hand, the lobar CMB count or the deep CMB count showed no correlation to the global, frontal, or occipital PiB uptake (Supplementary Figures 1 and 2). In addition, the ICH location (lobar/deep) showed no correlation to the global (p = 0.328) or regional PiB retention (frontal, p = 0.160; occipital, p = 0.272).

The results of the multivariable linear regression for global, frontal, and occipital PiB SUVR (using age, hypertension [yes/no], ICH days, ICH location [lobar/deep], CMB distribution [strictly lobar/mixed or deep], lobar CMB count, deep CMB count, and CMB ratio as potential predictors) are summarized in Table 2. Older age was associated with higher global and regional (frontal, occipital) amyloid retention. There were positive associations between CMB ratio and global or occipital amyloid burden, but not frontal amyloid burden.

The results of the logistic regression for positive PiB scan (SUVR > 1.15) (using age ≥ 55, hypertension, ICH days, lobar ICH, strictly lobar CMB, lobar CMB count, deep CMB count, and CMB ratio as potential predictors) are summarized in supplementary 2. CMBR remained an independent risk factor for positive PiB scan (odds ratio 6.018; 95% confidence interval 1.043–34.719; p = 0.045). Using CMBR to predict a diagnosis of CAA (CMBR > 2 as a positive value), the Az value, sensitivity, specificity, PPV, and NPV of the SUVR for positive PiB scan were 0.746, 63.6%, 88%, 82.4%, and 73.3%, respectively.

## Discussion

CMBs are defined as small, round, hypointense lesions that are visible on T2*-weighted gradient-recalled echo or SWI sequences^9^. It has been suggested that their topographical distribution indicates the presence and severity of underlying small vessel disease^9^. However, the presence of mixed CMBs distributed in both the lobar and deep regions still represents a clinical challenge. In the present study, cerebral amyloid burden in patients with mixed CMB distribution was higher than that in patients with only deep CMBs, but lower than that in patients with only lobar CMBs. Amyloid deposition, especially in the occipital lobe, was positively correlated with the ratio of lobar to deep CMB count. Collectively, these results show that for patients with CMBs in mixed location (i.e., in both the lobar and deep regions), a predominantly lobar distribution of CMBs is likely to be associated with underlying amyloid angiopathy.

The histopathological studies of CMBs have demonstrated hemosiderin-laden macrophages or hemosiderin deposits in the perivascular space^22^,^23^. HA (lipofibrohyalinosis) and CAA are the dominant changes for the vessels adjacent to CMBs^22^,^23^,^24^. Using PiB scans, which provides the advantage of *in vivo* detection of amyloid deposition in patients with CAA^17^, lobar CMBs have been well correlated with brain amyloid burden. For example, Yates *et al*. investigated 138 subjects without ICH using PiB PET and SWI scans, and discovered a strong association between PiB SUVR and lobar CMBs^25^. Another PiB PET study with longitudinal follow-up in CAA patients found that new lobar CMBs occur preferentially at sites of increased amyloid deposition^16^. Similarly, our study revealed a higher global and regional amyloid deposition in patients with exclusively lobar CMBs than in patients with exclusively deep CMBs.

Few studies have investigated the underlying vascular pathology of mixed CMBs. HA is believed to cause CMBs in both lobar and deep areas^22^,^26^,^27^, but whether CAA can cause deep CMBs is still controversial. Our study showed that patients with mixed CMBs were associated with higher deep CMB count than those with deep CMBs, suggesting a more severe underlying vasculopathy. Patients with mixed CMBs (compared to patients with deep CMBs) were older and more likely to have lobar ICH. Amyloid deposition was also higher in patients with mixed CMBs than in those with deep CMBs, indicating the possible difference in vascular pathology between these two types of CMBs. In addition, amyloid burden was lower in patients with mixed CMBs than in those with lobar CMBs. These results suggested that the pathogenesis of mixed CMBs may be heterogeneous and not completely attributable to CAA. Mixed vascular pathologies of CAA and HA were another possibility. We demonstrated that the amyloid burden was positively correlated with the ratio of lobar to deep CMB count after adjustment for age, ICH location and days, comorbid hypertension, CMB distribution and CMB count. On the other hand, amyloid burden was unrelated to lobar CMB count or deep CMB count. These findings suggest that the involvement and severity of both lobar and deep CMBs should be considered when determining the underlying vasculopathy in patients with mixed CMBs. Mixed CMBs may be associated with CAA if the CMBs are predominantly located in the lobar region.

The occipital lobe is most frequently and severely affected in CAA^28^,^29^. In previous studies using PiB PET in non-demented CAA patients, the amyloid deposits were found mainly in the occipital lobe^13^,^14^. In the present study, multivariable regression analysis showed an association of cerebral amyloid burden with CMB ratio and age. The high CMB ratio was related to the increase of amyloid burden in the occipital lobe but not in the frontal lobe. This result is compatible with the occipital lobe predominance of amyloid deposition in CAA, and further supports the role of CMB ratio in CAA diagnosis. On the other hand, older age was related to the increase of amyloid burden in both the occipital and frontal lobes, which may be due to age-related cortical amyloid deposition^30^.

According to the Boston criteria^31^, a diagnosis of possible or probable CAA requires at least one lobar, cortical, or cortico-subcortical hematoma. In the current study, there was no difference in PiB uptake between patients with lobar ICH and deep ICH, which suggested an equal amyloid burden in patients with different hematoma location. This result shows that the current etiological classification that depends on hematoma location might have a low accuracy. Recent studies have demonstrated that adding lobar CMB as a criterion may increase the sensitivity of CAA detection^32^,^33^. In our study, although there are regional associations between ICH and CMB locations, some discrepancies existed. Approximately 12.5% of patients with lobar type CMBs had deep ICH and 27.3% of patients with deep type CMBs had lobar ICH. Furthermore, 60% of our patients had mixed type CMBs, in which the underlying etiology is still an unanswered question. Our study showed that the CMB distribution pattern is a better predictor of CAA than the ICH location. Using CMBR > 2 as a cutoff value, the sensitivity and specificity of SUVR for predicting positive PiB scan were 63.6% and 88%, respectively. These results suggested that combining ICH location with CMB distribution pattern (i.e. CMBR) may help us to increase the diagnostic accuracy for detecting underlying vasculopathy in primary ICH patients.

This study had some limitations. First, the amyloid burden in ICH patients might be attributable to beta amyloid deposition in the cerebral blood vessels or in the brain parenchyma^34^. Differentiating between PiB retention patterns in CAA and in preclinical AD or mild cognitive impairment has not been well defined in previous studies. Although we evaluated the patient’s cognitive function retrospectively based on the reports from family members, and excluded patients with obvious dementia before ICH onset, the possibility of AD or mild cognitive impairment in some patients could not be totally ruled out. In addition, because there were significant age differences between patients with lobar, mixed and deep CMBs, age-related cortical amyloid deposition might also affect our result. Second, the majority of ICH patients in our study had hypertension regardless of CMB distribution. Whether hypertension and other vascular risk factors also accelerate amyloid deposition, as they do vascular cognitive impairment, remains to be confirmed in further studies^35^. Another concern is the undetermined nature of cerebellar CMB. In the current study, cerebellar CMB was classified as deep CMB because cerebellar hemorrhage has been considered of hypertensive cause in most of the cases^36^,^37^,^38^. However, cerebellar hemorrhage can also be associated with CAA^10^,^38^. It is possible that some the cerebellar CMBs were actually resulted from CAA. Furthermore, the sample size in this study was relatively small, and there was possible selection bias because patients who had poor functional outcome or who are intolerant to the exams were less likely to be recruited for participation in this study. In addition, there was wide range from the ICH event to the brain image examination, causing difficulties to determine the underlying vasculopathy for some of our patients with ICH. Future large studies are necessary to validate the present results.

In conclusion, this study offers evidence that cerebral amyloid deposition occurs not only in patients with exclusively lobar CMBs, but also in patients with both lobar and deep CMBs, and that the ratio of lobar to deep CMB count is positively correlated with cerebral amyloid burden. These findings support the diagnosis of CAA in patients meeting the Boston criteria but also having deep CMBs. Patients with ICH should regularly receive SWI or T2*-weighted MRI to predict amyloid deposition. A lobar-only or lobar-predominant distribution of CMBs increases the possibility of a diagnosis of CAA in non-demented patients with primary ICH.

## Additional Information

**How to cite this article:** Tsai, H.-H. *et al*. Correlation of Cerebral Microbleed Distribution to Amyloid Burden in Patients with Primary Intracerebral Hemorrhage. *Sci. Rep.* **7**, 44715; doi: 10.1038/srep44715 (2017).

**Publisher's note:** Springer Nature remains neutral with regard to jurisdictional claims in published maps and institutional affiliations.

## Supplementary Material

Supplementary Files

## Figures and Tables

**Table 1:** Demographics in patients with different CMB pattern.

	Lobar (n = 8)	Mixed (n = 28)	Deep (n = 11)	*p* value
Age, y	79.9 ± 12.5	65.9 ± 12.3	58.5 ± 14.0	**0.009**
Hypertension	3 (37.5%)	22 (78.6%)	9 (81.8%)	0.063
Diabetes	2 (25%)	4 (14.3%)	2 (25%)	0.757
Dyslipidemia	1 (12.5%)	6 (21.4%)	4 (36.4%)	0.561
Site of intracerebral hemorrhage				**0.035**
Lobar	7 (87.5%)	15 (53.6%)	3 (27.3%)	
Deep/infratentorial	1 (12.5%)	13 (46.4%)	8 (72.7%)	
SMASH-U				
Amyloid angiopathy	6 (75%)	12 (42.9%)	2 (18.2%)	**0.019**
Hypertension	0 (0%)	13 (46.4%)	6 (54.5%)	**0.022**
Undetermined	2 (25%)	3 (10.7%)	3 (27.3%)	0.504
Mini-Mental State Examination*	19.7 ± 8.4	24.1 ± 6.8	25.9 ± 4.6	0.188
Clinical Dementia Rating ≤ 0.5	8 (100%)	28 (100%)	11 (100%)	1
CMB count				
Lobar	11.4 ± 11.2	13.8 ± 15.5		0.434
Deep		11.0 ± 12.0	2.0 ± 1.3	**0.001**

Values are mean ( ± standard deviation) or number (percentage). *Not performed in 5 patients due to aphasia. CMB: cerebral microbleed; ICH: intracerebral hemorrhage.

**Table 2:** Results of multivariable analysis for PiB uptake.

	Global PiB SUVR		Frontal PiB SUVR		Occipital PiB SUVR	
	β (SE)	*p* value	β (SE)	*p* value	β (SE)	*P* value
Age	0.008 (0.003)	0.007	0.009 (0.003)	0.010	0.007 (0.002)	0.008
Hypertension (yes/no)	0.076 (0.081)	0.354	0.063 (0.094)	0.507	0.047 (0.068)	0.501
Days after ICH	0.000 (0.000)	0.226	0.000 (0.000)	0.454	0.000 (0.000)	0.279
ICH location (lobar/deep)	−0.005 (0.072)	0.948	−0.009 (0.084)	0.919	0.030 (0.061)	0.628
Strictly lobar CMB (yes/no)	0.429 (0.273)	0.125	0.463 (0.319)	0.155	0.343 (0.232)	0.148
CMB count						
Lobar	−0.003 (0.003)	0.373	−0.003 (0.004)	0.433	−0.002 (0.003)	0.543
Deep	0.003 (0.004)	0.358	0.003 (0.004)	0.449	0.001 (0.003)	0.740
CMB ratio*	0.011 (0.005)	0.028	0.011 (0.006)	0.055	0.009 (0.004)	0.031

β: regression coefficient; CMB: cerebral microbleed; ICH: intracerebral hemorrhage; SE: standard error; PiB: ^11^C-Pittsburgh Compound B; SUVR: standardized uptake value ratio. *CMB ratio is defined as the lobar CMB count divided by the deep CMB count.
