# Supplementary material for: Correlation of Cerebral Microbleed Distribution to Amyloid Burden in Patients with Primary Intracerebral Hemorrhage
Source: Sci Rep. 2017 Mar 17;7:44715. doi: 10.1038/srep44715 (PMC5356186; doi:10.1038/srep44715)
Supplement: Supplementary Files [file srep44715-s1.pdf]

# Correlation of Cerebral Microbleed Distribution to Amyloid Burden in Patients with Primary Intracerebral Hemorrhage

Hsin-Hsi Tsai, MD[1]; Li-Kai Tsai, MD, PhD[1]; Ya-Fang Chen, MD[2]; Sung-Chun Tang, MD, PhD[1]; Bo-Ching Lee, MD[2]; Ruoh-Fang Yen, MD, PhD[3]; Jiann-Shing Jeng, MD, PhD[1]

[1] Departments of Neurology, National Taiwan University Hospital, Taipei, Taiwan

[2] Department of Medical Imaging, National Taiwan University Hospital, Taipei, Taiwan

[3] Department of Nuclear Medicine, National Taiwan University Hospital, Taipei, Taiwan

## Supplementary Figure Legends

**Supplementary Figure 1 Correlation of lobar cerebral microbleed count and amyloid burden**

The correlation of lobar cerebral microbleed (CMB) count to the global PiB standardized uptake value ratio (SUVR) (A), frontal PiB SUVR (B) and occipital PiB SUVR (C).

**Supplementary Figure 2 Correlation of deep cerebral microbleed count and amyloid burden**

The correlation of deep cerebral microbleed (CMB) count to the global PiB standardized uptake value ratio (SUVR) (A), frontal PiB SUVR (B) and occipital PiB SUVR (C).

**Supplementary Table 1: Comparison of other image markers in patients with different CMB pattern**

| | **Lobar (n=8)** | **Mixed (n=28)** | **Deep (n=11)** | ***p* value** |
|---|---|---|---|---|
| White matter score | 1.88 ± 0.84 | 2.18 ± 0.72 | 1.82 ± 0.75 | 0.320 |
| Cortical superficial siderosis | 3 (37.5%) | 3 (10.7%) | 0 (0%) | 0.064 |
| Enlarged perivascular space (number > 20) | | | | |
| Centrum Semiovale | 2 (25%) | 18 (64.3%) | 4 (36.4%) | 0.102 |
| Basal ganglia | 2 (25%) | 6 (21.4%) | 5 (45.5%) | 0.385 |

Values are mean (±standard deviation) or number (percentage).

**Supplementary Table 2: Results of Logistic regression for Positive PiB Scan (SUVR>1.15)**

| | **Odds Ratio** | ***95% CI*** | ***p* value** |
|---|---|---|---|
| Age ≥55 | 5.258 | 0.416-66.409 | 0.200 |
| Hypertension | 1.135 | 0.149-8.671 | 0.903 |
| Days after ICH | 1.000 | 0.999-1.001 | 0.844 |
| Lobar ICH | 0.052 | 0.002-1.001 | 0.068 |
| Strictly lobar CMB | 0.000 | 0.000-1.699 | 0.051 |
| CMB count | | | |
| Lobar | 0.968 | 0.880-1.065 | 0.507 |
| Deep | 1.056 | 0.960-1.161 | 0.261 |
| **CMB ratio*** | **6.018** | **1.043-34.719** | **0.045** |

CMB: cerebral microbleed; CI: Confidence interval; ICH: intracerebral hemorrhage; PiB: $^{11}$C-Pittsburgh Compound B; SUVR: standardized uptake value ratio.

*CMB ratio is defined as the lobar CMB count divided by the deep CMB count.

**Supplementary Figure 1**

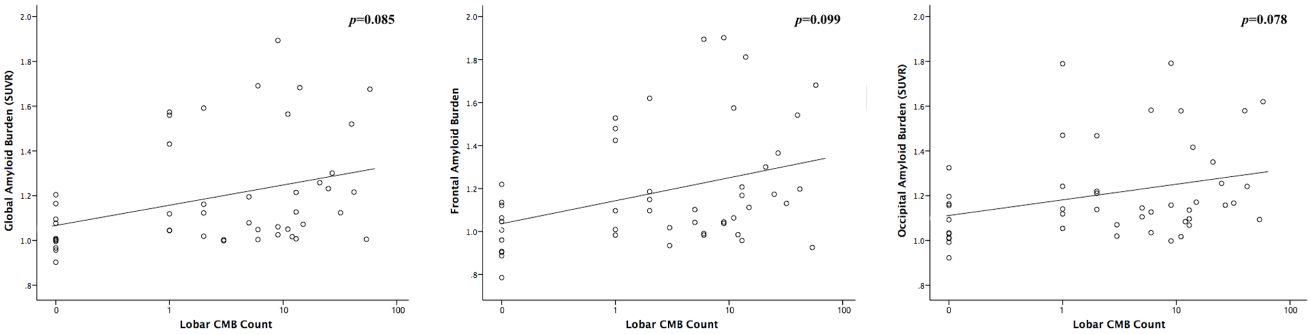
p=0.085
Global Amyloid Burden (SUVR)
Lobar CMB Count
p=0.099
Frontal Amyloid Burden
Lobar CMB Count
p=0.078
Occipital Amyloid Burden (SUVR)
Lobar CMB Count

**(A)** **(B)** **(C)**

**Supplementary Figure 2**

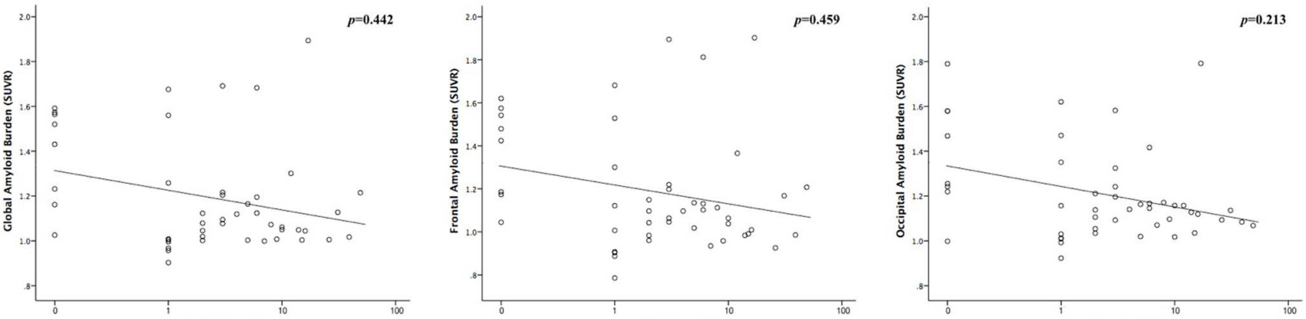
p=0.442
Global Amyloid Burden (SUVR)
Deep CMB Count
p=0.459
Frontal Amyloid Burden (SUVR)
Deep CMB Count
p=0.213
Occipital Amyloid Burden (SUVR)
Deep CMB Count

**(A)** **(B)** **(C)**
